# Tonsillar hypertrophy and prolapse in a child – is epiglottitis a predisposing factor for sudden unexpected death**?**

**DOI:** 10.1186/s12887-020-1927-3

**Published:** 2020-01-20

**Authors:** I. Nieuwoudt, P. Goussard, J. Verster, J. Dempers

**Affiliations:** 1 Department of Pathology, Division of Forensic Pathology, Faculty of Medicine and Health Sciences, Stellenbosch University and Tygerberg Hospital, Tygerberg, South Africa; 2 Department of Pediatrics and Child Health, Faculty of Medicine and Health Sciences, Stellenbosch University and Tygerberg Hospital, Tygerberg, PO Box 241, Cape Town, 8000 South Africa

**Keywords:** Tonsillitis, Tonsillar hypertrophy, Airway obstruction, Sudden and unexplained death in childhood, Sudden and unexplained death in infancy, Autopsy

## Abstract

**Background:**

Tonsillitis, with associated tonsillar hypertrophy, is a common disease of childhood, yet it is rarely associated with sudden death due to airway obstruction. Lethal complications involving the inflamed tonsils include haemorrhage, retropharyngeal abscess and disseminated sepsis.

**Case presentation:**

We report on a case of sudden and unexpected death in an 8-year-old female who was diagnosed with and treated for tonsillitis. The child was diagnosed with acute tonsillitis 2 days prior to her collapse and was placed on a course of oral antibiotics. There were no signs of upper or lower airway obstruction. She was found to be unresponsive by her caregiver and gasping for air in her bed in the early hours of the second morning after the start of treatment. Autopsy showed massive and symmetrically enlarged palatine tonsils. The tonsils filled the pharynx almost completely. The epiglottis and laryngeal mucosa at the base of the epiglottis in the vicinity of the aryepiglottic membrane and the superior aspect of the larynx displayed red-purple discoloration, with mucosal swelling and edema. Histological examination of the palatine tonsils revealed prominent lymphoid hyperplasia, but no evidence of acute inflammation.

**Conclusion:**

Palatine tonsillar hypertrophy in infants is a common feature of both viral and bacterial tonsillitis and has been postulated as a possible risk factor for Sudden and Unexplained Death in Infancy (SUDI), based on the theory of mechanical impediment of breathing by narrowing of the upper airway. The rounded shape of the tonsils may facilitate some airflow past the enlarged structures and hence protect against asphyxial death when the enlarged tonsils fill the laryngo-pharynx. Epiglottal and proximal laryngeal edema may play a more significant role in asphyxial unexpected deaths in cases of tonsillitis with tonsillar hypertrophy than previously suspected. This focusses the importance of careful examination of the epiglottis and proximal laryngeal mucosa, as part of a thorough examination of the laryngo-pharynx in cases of sudden death associated with tonsillar hypertrophy.

## Background

Tonsillitis with associated tonsillar hypertrophy is a common disease of childhood, yet it is rarely associated with sudden death due to airway obstruction.

Tonsillitis can be caused by viral or bacterial pathogens. Common viral pathogens resulting in acute pharyngitis include *Rhinovirus, Coronavirus, Adenovirus, Respiratory Syncytial Virus, Haemophilus influenza, Parainfluenza, Herpes Simplex virus, Coxsackie virus, Cytomegalovirus* as well as *Epstein Barr Virus*. Bacterial pathogens include mainly *Streptococcus pyogenes, Neisseria Meningitidis, Corynebacterium diphtheria, Chlamydia and Mycoplasma pneumonia* [1]. Lethal complications involving the inflamed tonsils include haemorrhage, retropharyngeal abscess formation and disseminated sepsis. Many of these are surgery related [2]. Massive haemorrhage may also occur due to erosion of the tonsillar vessels or adjacent larger vessels.

## Case presentation

We report on a case of sudden and unexpected death in an 8-year-old female who was diagnosed with and treated for tonsillitis. Tonsillar hypertrophy was present, and obstruction of the airway was suspected to be the mechanism of death. The child was diagnosed with acute tonsillitis 2 days prior to her collapse and was placed on a course of oral antibiotics (Cefpodoxime) and oral paracetamol at her local clinic, and sent home. She was previously well, with no history of chronic disease or history of allergy. Clinical examination at the time was not suggestive of upper or lower airway obstruction and she was not toxically sick. No bacterial or viral cultures or PCR testing was done, as she presented to a local clinic. During the course of the day preceding her death she was feverish and vomited, but she remained responsive. She was found to be unresponsive and gasping for air by her mother’s sister in her own bed in the early hours of the second morning after the start of the antibiotics. Resuscitation efforts at a local medical facility were unsuccessful and death was declared approximately 30 min after arrival at the facility.

A medico-legal autopsy was indicated under South African law as the death was classified as sudden and unexpected. The main external findings at autopsy were that the child was of normal weight and build for age. No fresh injuries or other signs of trauma were present. Both eyes were slightly sunken into the orbits. The tip of the tongue was clenched between the teeth.

**Fig. 1:**
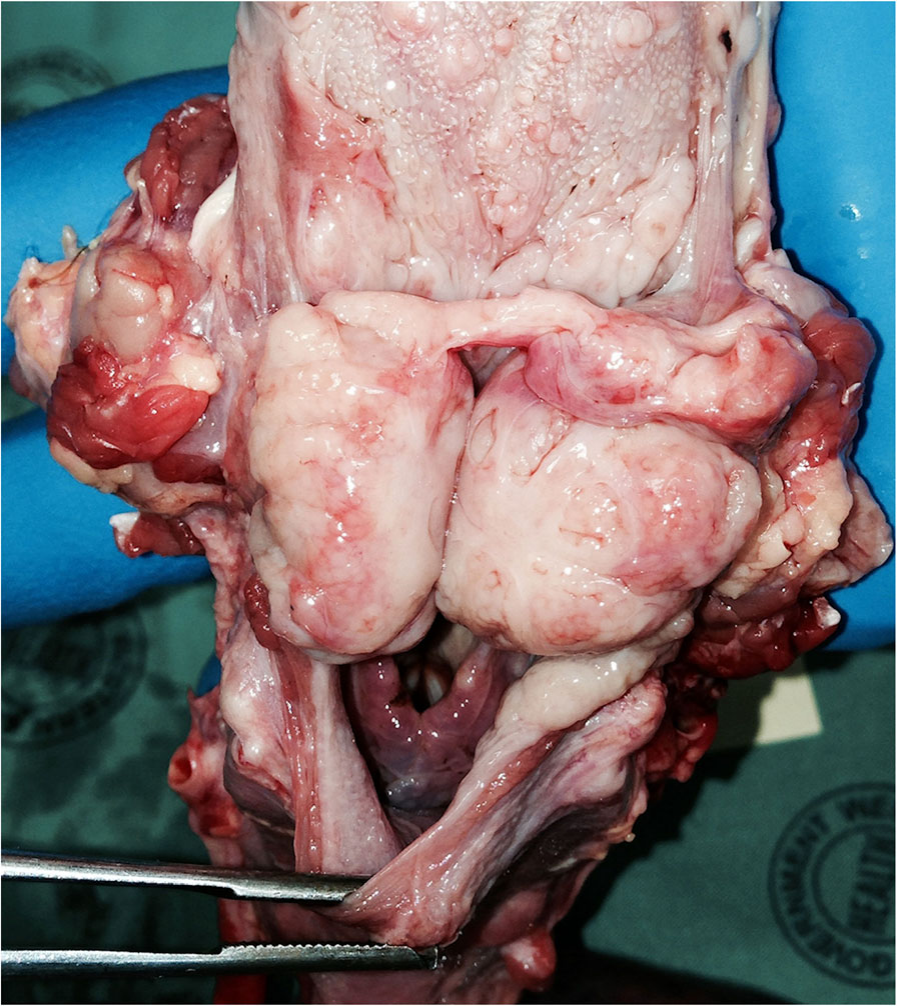
Laryngo-pharyngeal specimen after removal at autopsy with enlarged palatine tonsils and inflamed, edematous upper laryngeal mucosa

Internal examination revealed massive and symmetrically enlarged palatine tonsils (so-called kissing tonsils) with the right tonsil measuring 32 mm × 23 mm × 15 mm and left tonsil 25 mm × 21 mm × 12 mm. The tonsils filled the pharynx almost completely. The right tonsil appeared slightly more mobile at its point of attachment than the left, abutting against the epiglottis. No surface exudate, pseudo-membrane formation or parenchymal haemorrhage were present on the surface, or in the tonsils; the tonsils only appeared slightly hyperaemic. The epiglottis and laryngeal mucosa at the base of the epiglottis in the vicinity of the aryepiglottic membrane and the superior aspect of the larynx displayed red-purple discoloration, with mucosal swelling and edema (Fig. 1). The tonsils were removed with the laryngo-pharyngeal block to preserve the relative position of the structures to each other.

There were no macroscopic or microscopic signs of pathology in the lungs. Epicardial haemorrhages were present over the left ventricle of the heart, but there were no signs of congenital cardiac disease and the heart size was normal. The brain appeared full but did not exhibit signs of frank herniation. The organ weighed 1360 g (mean weight 1273 g) [3]. The lepto-meningeal membranes were not opacified.

**Fig. 2:**
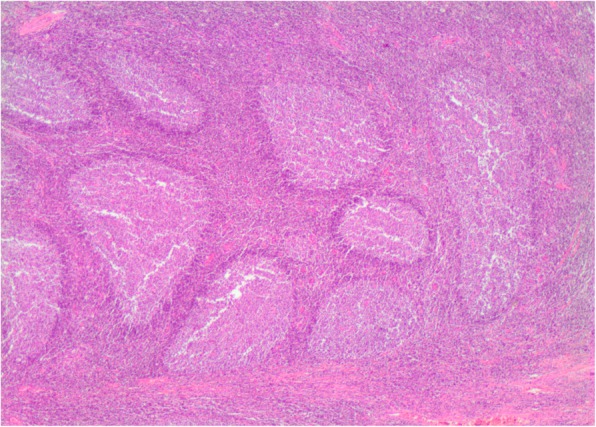
Histomicrograph illustrating tonsillar hypertrophy (Hematoxillin & Eosin stain; × 20)

**Fig. 3:**
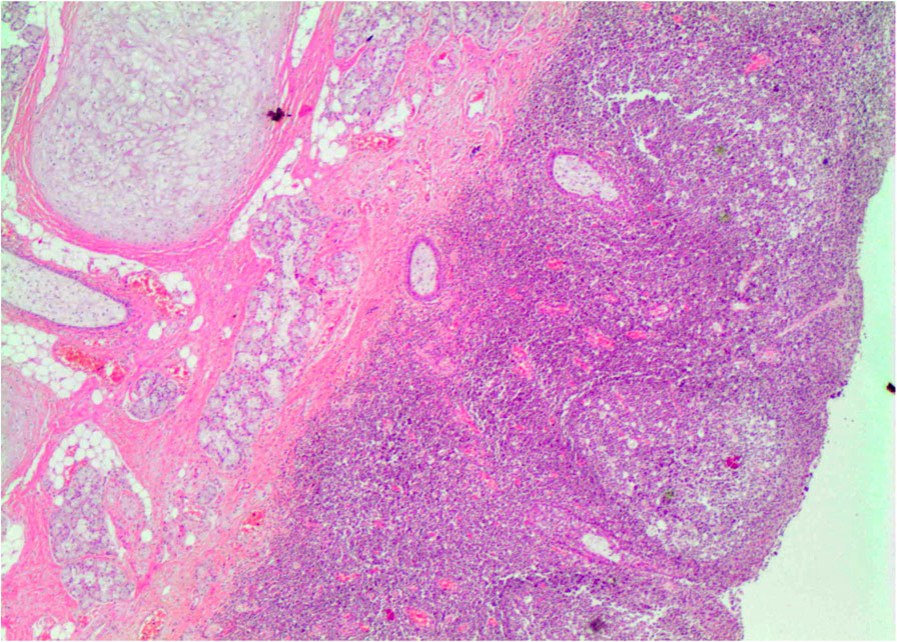
Histomicrograph illustrating dense epiglottal chronic inflammation and edema (Hematoxillin & Eosin stain; × 20)

Histological examination of the palatine tonsils revealed prominent lymphoid hyperplasia but no evidence of acute inflammation (Fig. 2). The epiglottis and mucosa at the upper margin of the larynx showed dense chronic inflammatory cell infiltration and edema, but again no signs of acute inflammation or pus were present (Fig. 3). There were no signs of pseudomembrane formation in either the upper or lower airways that could have caused fatal airway obstruction. No signs of haemorrhage were present.

Special stains and immunohistochemistry of the tonsillar tissue confirmed the normal cyto-architecture of reactive tonsillar hypertrophy. Special stains did not prove positive for fungi or other viruses, including *Epstein-Barr Virus and Cytomegalovirus*.

She was HIV negative and blood chemistry showed an elevated urea level of 13.4 mmol/ℓ (laboratory reference 1.4–5.7 mmol/ℓ) as well as an elevated creatinine level of 235 μmol/ℓ (laboratory reference 30–48 μmol/ ℓ). These levels are supportive of the clinical suspicion of mild dehydration and may have resulted from a combination of a hyperdynamic circulation, vomiting, anorexia and the mechanical difficulty in ingesting food and fluids, secondary to the tonsillar hypertrophy.

Complete autopsy, ancillary investigations and histological examination of the tissues did not reveal any other possible cause of death.

## Discussion and conclusions

The mortality of tonsillitis is low, and usually lethal complications result from surgical intervention. According to the literature, the mortality rate following tonsillectomy equates to 1/1000–1/27,000 [2]. Complications of acute tonsillitis resulting in death include airway compromise secondary to airway obstruction and septicaemia due to systemic progression of the infection [4]. Airway compromise usually results from bilateral tonsillar enlargement. This phenomenon has also been reported in a 19-month-old boy with unilateral tonsillar enlargement, where a pedunculated left palatine tonsil occluded the glottis [5].

.Palatine tonsillar hypertrophy in infants is a common feature of both viral and bacterial tonsillitis, and has been postulated by Suzuki et al. [6] as a possible risk factor for Sudden and Unexplained Death in Infancy (SUDI), based on the theory of mechanical impediment of breathing by narrowing of the upper airway. It was further suggested that the degree of hypertrophy of the palatine tonsils appeared to be sufficient to narrow the upper airway from one or both sides of the pharynx and that the palatine tonsils might narrow the upper airway at the level of the pharynx, dependant on and facilitated by the sleeping position of the infant. This theory is on the face of it logical, yet deaths occurring as a result of uncomplicated, often significant infective tonsillar enlargement *without* surgical intervention are rare, or at the very least underreported. If enlarged tonsils can easily impact into the pharyngeal orifice, it is therefore quite surprising that deaths as a result of airway occlusion are apparently so rare.

Of importance to consider is also the relative size of the tonsils. The right palatine tonsil measured 32 mm (h) × 23 mm (w) and left tonsil 25 mm (h) × 21 mm (w). These dimensions need to be evaluated against the measured height and width of the tonsils as reported by Jong Hwan Wang et al. [7]. The subjective tonsil height in children between the ages of 3 years to 17 years old vary between 16,7 mm – 33,1 mm and the subjective tonsil width in this age group vary between 9,6 mm to 22,2 mm; however these subjective tonsillar sizes do not always correlate well with the actual tonsillar volume measured after tonsillectomy.

**Fig. 4:**
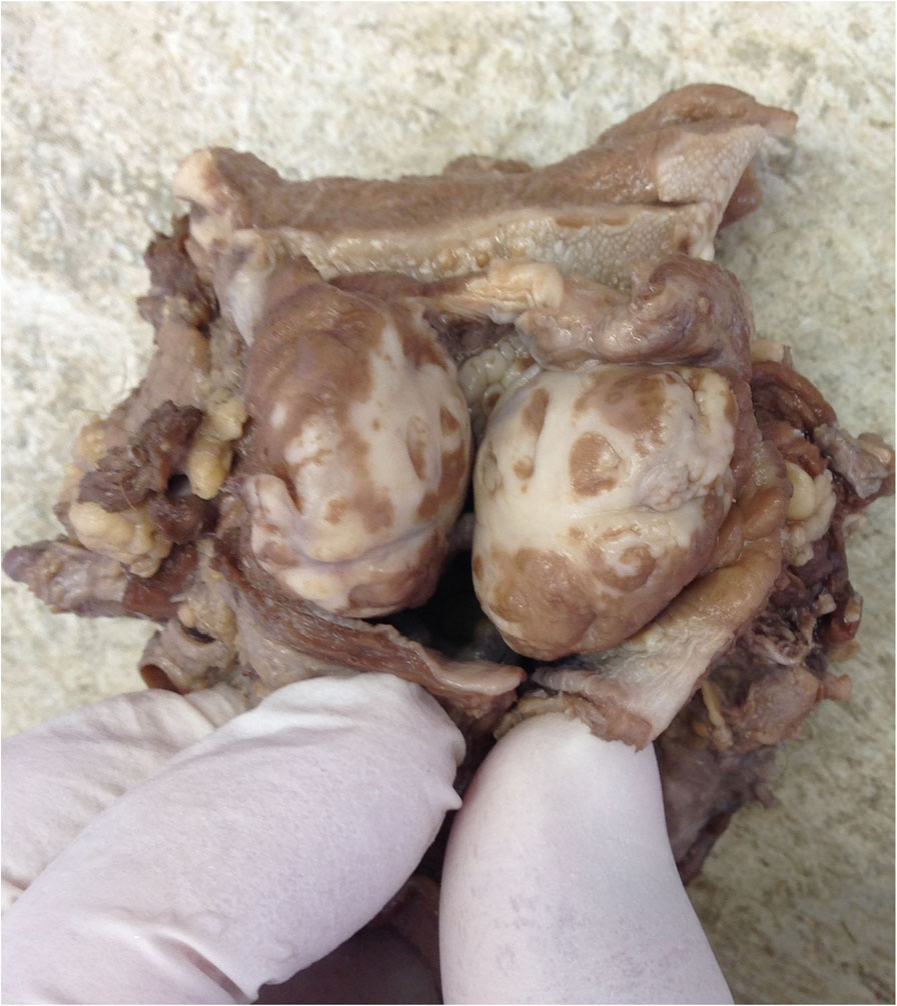
Formalin fixed laryngo-pharyngeal specimen with manual approximation of the posterior incised edges, illustrating the conduit in the posterior laryngo-pharynx that may facilitate airflow past the enlarged tonsils

We postulate that the rounded shape of both hypertrophied tonsils in the pharynx may play a protective role with regards to the maintenance of a functional conduit through which respiration may be retained in the anterior, but apparently most prominently posterior aspect of the pharynx. As the tonsils protrude into the lumen of the pharynx, their rounded, expanded shape assures the formation of a triangular opening between the postero-medial aspects of the tonsils, and the posterior wall of the pharynx. This is clearly apparent on approximation of the incised posterior edges of the pharyngeal tissue block, not only in the fresh specimen, but also in the formalin fixed specimen. In our case, approximation of the pharyngeal ring and reconstitution of the enlarged tonsils to its in situ position results in a clearly identifiable opening measuring approximately 5 mm × 7 mm, even with the tonsils touching in the midline (Fig. 4). This aperture appears to be large enough to allow at least some air movement, when compared to the size of the pharyngeal aperture at the level of the cricoid cartilage in normal children. The epiglottis – a structure that is inconveniently located antero-inferior to this aperture formed by the posterior margins of the tonsils, can conceivably cause narrowing at this level if enlargement and edema of this structure and the surrounding superior laryngeal opening causes expansion of the tissue. In our case, the epiglottis and proximal laryngeal ring was indeed oedematous and exhibited signs of epiglottitis on histology.

Whereas there can be no doubt that massive tonsillar hypertrophy can in extreme cases cause critical airway obstruction, we postulate that the epiglottis may play a more significant role in the pathogenesis of sudden unexpected airway obstruction associated with tonsillar hypertrophy than previously suspected.

Byard et al. [5] emphasizes the need to examine the upper aerodigestive tract at autopsy in all age groups, not only early childhood, as such lesions may not produce marked symptoms and signs prior to lethal airway occlusion.

## Conclusions

Sudden death following acute tonsillitis has been reported in the literature, but limited information is available on the possible mechanisms of death that may occur in cases of sudden and unexpected death associated with tonsillar pathology. Our case represents one of most likely viral tonsillitis with significant enlargement of the palatine tonsils. We postulate that airway obstruction in this case resulted from tonsillar hypertrophy, as well as the contributory effect of epiglottitis, which further compromised the upper airway as a result of mucosal swelling and edema.

We suggest that particular attention should be paid to the epiglottis – both macro and microscopically, as concomitant epiglottal edema may very well offer an explanation for the sudden and unexpected collapse and death of children with tonsillitis and associated tonsillar hypertrophy.

## Data Availability

Material is available.

## Abbreviations

Fig: Figure
SUDI: Sudden and Unexplained Death in Infancy
